# Cardiac CTA image quality of adaptive statistical iterative reconstruction-V versus deep learning reconstruction “TrueFidelity” in children with congenital heart disease

**DOI:** 10.1097/MD.0000000000031169

**Published:** 2022-10-21

**Authors:** Kun Hee Kim, Ki Seok Choo, Kyoung Jin Nam, Kyeyoung Lee, Jae-Yeon Hwang, ChanKue Park, Woo Jung Yang

**Affiliations:** a Department of Radiology, Pusan National University School of Medicine and Research Institute for Convergence of Biomedical Science and Technology, Pusan National University Yangsan Hospital, Yangsan-si, Gyeongsangnam-do, Korea; b Barunmom Rehabilitation Medicine, Busanjin-gu, Busan, Korea.

**Keywords:** deep learning, image quality enhancement, image reconstruction, pediatric CT angiography

## Abstract

Several recent studies have reported that deep learning reconstruction “TrueFidelity” (TF) improves computed tomography (CT) image quality. However, no study has compared adaptive statistical repeated reconstruction (ASIR-V) using TF in pediatric cardiac CT angiography (CTA) with a low peak kilovoltage.

**Objective:**

This study aimed to determine whether ASIR-V or TF CTA image quality is superior in children with congenital heart disease (CHD).

**Materials and methods:**

Fifty children (median age, 2 months; interquartile range, 0–5 months; 28 men) with CHD who underwent CTA were enrolled between June and September 2020. Images were reconstructed using 2 ASIR-V blending factors (80% and 100% [AV-100]) and 3 TF settings (low, medium, and high [TF-H] strength levels). For the quantitative analyses, 3 objective image qualities (attenuation, noise, and signal-to-noise ratio [SNR]) were measured of the great vessels and heart chambers. The contrast-to-noise ratio (CNR) was also evaluated between the left ventricle and the dial wall. For the qualitative analyses, the degree of quantum mottle and blurring at the upper level to the first branch of the main pulmonary artery was assessed independently by 2 radiologists.

**Results:**

When the ASIR-V blending factor level and TF strength were higher, the noise was lower, and the SNR was higher. The image noise and SNR of TF-H were significantly lower and higher than those of AV-100 (*P* < .01), except for noise in the right atrium and left pulmonary artery and SNR of the right ventricle. Regarding CNR, TF-H was significantly better than AV-100 (*P* < .01). In addition, in the objective assessment of the degree of quantum mottle and blurring, TF-H had the best score among all examined image sets (*P* < .01).

**Conclusion:**

TF-H is superior to AV-100 in terms of objective and subjective image quality. Consequently, TF-H was the best image set for cardiac CTA in children with CHD.

## 1. Introduction

Cardiac computed tomography (CT) angiography (CTA) plays an important role in the diagnosis and treatment of congenital heart disease (CHD) in the pediatric population.^[1]^ Compared with other imaging modalities, cardiac CTA describes a more intuitive anatomical structure and helps in preoperative planning for surgeons; therefore, it prevents postoperative complications because it can be used to evaluate the airway and major vessels.^[2–7]^

CHD has been diagnosed prenatally in most recent cases, and cardiac CTA evaluation is performed immediately after birth. In other words, most CT scans are performed in newborns; therefore, it is very important to reduce radiation exposure as much as possible. The development of a CT protocol that improves image quality even at lower voltages is an issue in the pediatric population. As a dose-reduction technique, various reconstruction methods of 3D data from raw projection data have been highlighted to adequately treat image noise and artifacts.^[8]^

The conventional filtered back projection (FBP) algorithm has limitations for low-dose scans: it increases noise and produces aliasing degradation and streaky artifacts.^[9,10]^ Iterative reconstruction (IR), including adaptive statistical iterative reconstruction-V (ASIR-V), was developed to overcome the limitations of FBP.^[10–12]^ IR succeeded in reducing the radiation dose with denoising.^[12]^ However, in many studies, it has been proven that IR-based image reconstruction produces uncomfortable image textures (plastic like, paint-brushed, blurry, blotchy, or oversmooth) for radiologists.^[13–15]^

Deep learning–based image reconstruction (DLR), including TrueFidelity (TF), is a technology that introduces deep convolutional neural networks. Based on the high-dose low-noise data set, it trained production of reconstruction images of the low-dose data with relatively low-noise. Therefore it brings out image noise reduction and restores preferred noise texture, leading to improved objective and subjective image quality.^[16,17]^ In addition, DLR can compensate for the drawback of IR in several previous studies.^[18–21]^

Recently, many studies have reported that CT image reconstruction using DLR provides better image quality than IR.^[21–24]^ In the current study, the image quality between ASIR-V and TF was compared in terms of attenuation, noise, signal-to-noise ratio (SNR), and contrast-to-noise ratio (CNR) in a pediatric cardiac CTA performed at a very low tube voltage of 70 kVp. Also, availability of 70kVp is quite useful in reducing the radiation dose and increasing the iodine contrast-to-noise ratio in young children with CHD.^[4,25]^ The purpose of this study was to determine the best cardiac CTA image set between IR “ASIR-V” and deep learning reconstruction “TF” in children with CHD.

## 2. Materials and Method

### 2.1. Study population

This study was a retrospective study, and we received approval from the institutional review board with a waiver for informed consent. The inclusion criterion was pediatric patients who were suspected of having CHD underwent cardiac CTA (Revolution CT, GE Healthcare) and had both IR (ASIR-V) and DLR (TF) in reconstructed images. The presence of ambiguous anatomical borders in the patients was an exclusion criterion.

### 2.2. CT protocol

All CT examinations underwent single-beat contrast-enhanced cardiac CTA during free-breathing with prospective ECG triggering at 45% of the R-R interval on a 512-slice CT scanner (Revolution CT, GE Healthcare, Waukesha, WI). All patients was sedated to achieve optimal imaging with minimizing motion artifacts by using hydroxyzine (1 mg/kg, orally) and midazolam (0.2 mg/kg, orally). The CT imaging parameters were as follows: smart Z-coverage (4, 8, 10, 12, 14, or 16 cm was used depending on the patient’s body size), 70 kV using kV Assist and ECG dose modulation (noise index: 15), and 280 ms gantry rotation time. Slice thickness and slice interval were both applied at 0.625 mm. The CT dose index estimation was obtained using a standard 32 cm phantom. All patients were injected with 2 mL/kg of iodinated contrast medium (iohexol; iobrix-300, Taejoon Pharmaceutical, Seoul, Korea) at a rate of 1.0 mL/s. The lower extremity vein was preferred to reduce image artifacts from the great vessels. A bolus tracking method was selected to evaluate the cardiac chamber and great vessels. A region of interest (ROI) was placed on the left ventricle (LV), and image acquisition was started 2 seconds after signal attenuation reached the predefined threshold of 150 Hounsfield units (HU). The scan range was adjusted from apex to cardiac base. All CT images were reconstructed using ASIR-V with blending factors of 80% and 100% (AV-80 and AV-100) and low-, medium-, and high-strength DLR TF (TF-L, TF-M, and TF-H, respectively).

### 2.3. Objective analysis

**Figure 1.**
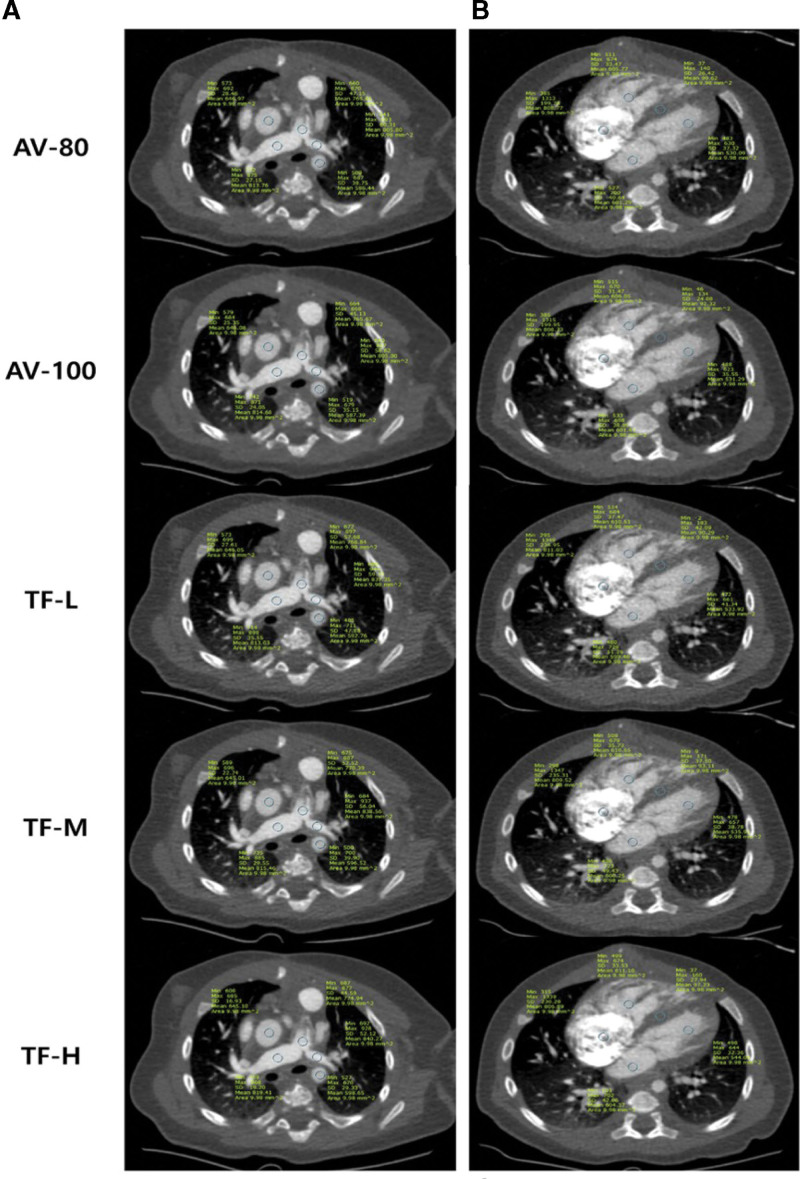
Axial contrast-enhanced CT images from a 1-month-old female patient with atrial and ventricular septal defect, at the visible level of the pulmonary trunk, right pulmonary artery, left pulmonary artery and 4-chamber view. ROI was measured for each reconstruction image of (a) AV-80, (b) AV-100, (c) TF-L, (d) TF-M, and (e) TF-H, then a total of 10 structures, namely, the left atrium, left ventricle, right atrium, right ventricle, pulmonary trunk, left pulmonary artery, right pulmonary artery, ascending aorta, descending aorta, and interventricular septum, were obtained. AV-80 and AV-100 = ASIR-V with blending factors of 80% and 100%, respectively; TF-L, TF-M, and TF-H = TrueFidelity with low, medium, and high strength levels, respectively. CT = computed tomography, ROI = region of interest.

Two radiologists with 2 and 20 years of experience conducted an objective image analysis of the axial images of the cardiac CTA in random order by consensus (Fig. 1).

They were blinded to patient information during the analysis with using PACS’s random order system. ROI was obtained from the pulmonary trunk, left pulmonary artery, right pulmonary artery, ascending aorta, and descending aorta at the axial image level of the first branch of both main pulmonary arteries. The left atrium, LV, right atrium, right ventricle, and interventricular septum were also obtained in the 4-chamber view (Fig. 1). ROI was measured with a circle area of 8 to 10 mm^2^, and the values of attenuation, standard deviation which represented nosie, and SNR of each lesion were derived. Furthermore, the CNR between the LV blood pool and the myocardial wall was evaluated using the following formula:


$$\begin{array}{l} SNR\;=\;target\;HU/\;target\;SD. \end{array}$$

The CNR between the LV blood pool and the myocardial wall was calculated as follows:


$$\begin{array}{l} CNR\;=\;\left(LV\;blood\;pool\;HU\;\;LV\;myocardium\;wall\;HU\right)/\;LV\;SD \end{array}$$

### 2.4. Subjective analysis

Each radiologist independently evaluated the quantum mottle and blurring of reconstructed images from ASIR-V (AV-80 and AV-100) and DLR (TF-L, TF-M, and TF-H) at the axial image level of the first branch of the left and right pulmonary arteries. The patient’s information was blinded, and the images were executed in a random order. The standard window level and width values were set to WW 1600 and WL 300, respectively.

All reconstruction images were divided into grades 0 to 3 as follows: quantum mottle (grade 0, diffuse homogeneous without mottle; grade 1, mild mottle; grade 2, moderate mottle; and grade 3, diffuse inhomogeneous with severe mottle) and blurring (grade 0, no blurring; grade 1, mild blurring; grade 2, moderate blurring; and grade 3, severe blurring). Subsequently, the final image quality was evaluated by adding the quantum mottle and blurring values.^[21,25]^ The lower the score, the better the subjective image quality.

### 2.5. Estimation of radiation dose

To evaluate the effective radiation dose, the estimated CT dose index volume and dose-length product (DLP) were collected from the dose report after the CT scan. The radiation dose was calculated according to the modified International Commission on Radiological Protection Publication 103. The conversion factor for DLP is specified by age and sex.^[26,27]^ To calculate the effective dose in this study, the DLP was multiplied by conversion factors of 0.029 and 0.019 for male patients aged 1 month and 2 months to 1 year, respectively. In female patients, conversion factors of 0.052 and 0.033 were used for those aged 1 month and 2 months to 1 year, respectively.

### 2.6. Statistical analysis

For statistical analysis, statistical software SPSS version 26.0 (IBM) was used. To compare the image sets, objective and subjective analysis were performed using ANOVA with the Bonferroni post hoc test. To calculate inter-reader reliability for subjective analysis, we calculated the intraclass correlation coefficients, and the difference between the readers was measured using kappa (κ). The statistical range of κ values was interpreted using the following criteria: <0.20, poor; 0.21 to 0.40, fair; 0.41 to 0.60, moderate; 0.61 to 0.80, good; and 0.81 to 1.00, excellent. Statistical significance was set at *P* < .05.

## 3. Result

From June to September 2020, 52 pediatric patients with CHD fulfilled inclusion criterion. Among them, 2 patients with situs inversus and single ventricle were excluded because of their ambiguous anatomical borders.

Finally, 50 patients were included in this study: 28 were male (56%) and 22 were female (44%). The median age of the children was 2 months (interquartile range: 0–5 months). These patients were all diagnosed with complex CHD: Tetralogy of Fallot, n = 15; coarctation of the aorta, n = 9; atrial and ventricular septal defect, n = 4; total anomalous pulmonary venous return, n = 4; vascular ring, n = 4; atrial septal defect, n = 3; hypoplastic right heart syndrome, n = 3; truncus arteriosus, n = 2; double outlet right ventricle, n = 2; hypoplastic left heart syndrome, n = 2; Taussig–Bing anomaly, n = 1; and transposition of the great arteries, n = 1.

### 3.1. Objective analysis

**Figure 2.**
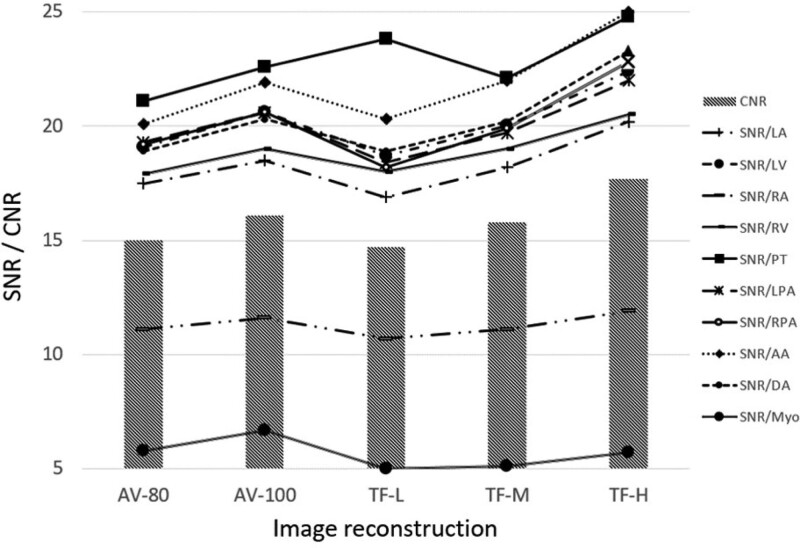
Schematic of the SNR of 10 anatomical structures and the CNR between LV blood pool and LV myocardial wall. The overall SNR values of TF-H was higher than that of AV-100 (*P* < .01). Regarding CNR, TF-H was significantly better than AV-100 (*P* < .01). AA = ascending aorta, AV-80 and AV-100 = ASIR-V with blending factors of 80% and 100%, respectively, CNR = contrast-to-noise ratio, DA = descending aorta, LA = left atrium, LPA = left pulmonary artery, LV = left ventricle, Myo = myocardium, PT = pulmonary trunk, RA = right atrium, RPA = right pulmonary artery, RV = right ventricle, SNR = signal-to-noise ratio, and TF-L, TF-M, and TF-H = TrueFidelity with low, medium, and high strength levels, respectively.

Table 1 and Figure 2 show a summary of the results of the objective analysis. The standard deviation values of the HU reflecting the noise were lower, at increased level of the ASIR-V blending factor (AV-80 < AV-100) or the TF strength (TF-L < TF-M < TF-H) in all 10 anatomical structures. Likewise, the SNR values were higher in the same order.

**Table 1:** Quantitative analysis.

	AV 80	AV 100	TF-L	TF-M	TF-H	*P*
LA						
HU	584.3^a^ ± 233.9	584.2^a^ ± 233.9	584.8^a^ ± 235.0	585.1^a^ ± 235.2	585.5^a^ ± 235.5	.327
SD	34.6^c^ ± 8.9	32.6^b^ ± 9.2	36.2^d^ ± 9.4	33.6^b,c^ ± 9.1	30.2^a^ ± 8.7	<.001
SNR	17.5^a,b^ ± 7.4	18.5^c^ ± 7.6	16.9^a^ ± 7.5	18.2^b,c^ ± 7.9	20.2^d^ ± 8.4	<.001
LV						
HU	579.8^a^ ± 225.7	579.9^a^ ± 225.7	580.6^a^ ± 226.4	580.8^a^ ± 226.5	580.9^a^ ± 226.6	.20
SD	31.5^c,d^ ± 11.3	29.6^b^ ± 11.7	32.6^d^ ± 11.4	30.6^b,c^ ± 11.2	27.4^a^ ± 10.7	<.001
SNR	19.1^a,b^ ± 6.3	20.6^c^ ± 6.8	18.7^a^ ± 7.0	20.0^b,c^ ± 7.5	22.4^d^ ± 8.4	<.001
RA						
HU	665.9^a^ ± 375.9	665.9^a^ ± 375.8	666.8^a^ ± 377.1	666.9^a^ ± 377.2	667.1^a^ ± 377.5	.052
SD	74.2^c^ ± 61.2	72.8^b^ ± 61.9	76.2^d^ ± 61.7	74.7^c^ ± 61.8	72.0^a^ ± 62.1	<.001
SNR	11.1^b^ ± 4.7	11.6^c^ ± 5.0	10.7^a^ ± 4.5	11.1^b^ ± 4.7	11.9^d^ ± 5.2	<.001
RV						
HU	622.7^a^ ± 286.0	622.6^a^ ± 286.0	623.3^a^ ± 286.4	623.5^a^ ± 286.4	623.6^a^ ± 290.2	.361
SD	37.6^b,c^ ± 20.1	35.9^b^ ± 20.5	38.2^c^ ± 42.6	36.4^d^ ± 20.6	33.5^a^ ± 20.5	<.001
SNR	17.9^a^ ± 6.9	19.0^b^ ± 7.2	18.0^a^ ± 7.6	19.0^b^ ± 8.1	20.5^c^ ± 9.7	<.001
PT						
HU	615.7^b^ ± 302.2	614.8^a^ ± 302.1	616.4^c^ ± 302.5	616.6^c^ ± 302.3	616.3^c^ ± 302.0	.002
SD	31.7^c^ ± 11.3	29.7^b^ ± 11.5	33.6^d^ ± 11.5	31.2^b,c^ ± 11.1	27.8^a^ ± 10.2	<.001
SNR	19.5^a^ ± 6.6	20.9^b^ ± 6.8	18.8^a,b,c^ ± 6.9	20.2^a,b^ ± 7.3	22.5^c^ ± 8.0	<.001
LPA						
HU	592.8^b^ ± 266.9	592.2^b^ ± 267.0	596.2^a^ ± 268.3	596.3^a^ ± 268.3	596.4^a^ ± 268.3	<.001
SD	31.4^c^ ± 10.6	29.7^b^ ± 10.8	33.5^d^ ± 10.1	31.4^c^ ± 9.9	28.4^a^ ± 9.7	<.001
SNR	19.3^b^ ± 6.5	20.6^c^ ± 6.9	18.4^a^ ± 7.0	19.7^b,c^ ± 7.4	22.0^d^ ± 8.4	<.001
RPA						
HU	595.2^a^ ± 270.9	595.2^a^ ± 270.9	597.2^b^ ± 272.7	597.5^b^ ± 272.5	598.2^c^ ± 272.5	<.001
SD	32.2^c^ ± 13.3	30.4^b^ ± 13.5	34.5^d^ ± 13.8	32.0^b,c^ ± 13.3	28.3^a^ ± 12.5	<.001
SNR	19.2^b^ ± 7.0	20.6^c^ ± 7.4	18.2^a^ ± 7.8	19.9^b,c^ ± 8.6	22.8^d^ ± 10.1	<.001
AA						
HU	587.9^a^ ± 245.2	594.7^b^ ± 234.4	595.8^c^ ± 234.7	596.1^d^ ± 234.7	596.1^d^ ± 234.5	.005
SD	31.2^c,d^ ± 11.4	29.2^b^ ± 11.6	32.4^d^ ± 12.6	30.2^b,c^ ± 12.5	27.0^a^ ± 12.2	<.001
SNR	20.1^a^ ± 8.3	21.9^b^ ± 8.7	20.3^a^ ± 9.4	22.0^b^ ± 10.3	25.0^c^ ± 11.7	<.001
DA						
HU	588.5^a^ ± 229.0	588.5^a^ ± 229.0	591.1^b^ ± 230.1	591.1^b^ ± 229.9	591.0^b^ ± 229.2	<.001
SD	32.2^c^ ± 10.2	30.3^b^ ± 10.5	32.7^c^ ± 9.3	30.7^b^ ± 9.6	27.0^a^ ± 9.5	<.001
SNR	18.9^a^ ± 6.4	20.3^b^ ± 6.8	18.9^a,b^ ± 7.4	20.2^b^ ± 7.5	23.3^c^ ± 8.9	<.001
Myocardium						
HU	119.5^b^ ± 143.4	119.6^b^ ± 143.4	118.6^a^ ± 143.2	118.7^a^ ± 143.2	118.4^a^ ± 143.1	.012
SD	22.9^b^ ± 6.2	20.2^a^ ± 6.1	27.5^d^ ± 8.0	25.9^c^ ± 7.3	23.0^b^ ± 6.4	<.001
SNR	5.8^c^ ± 8.6	6.7^d^ ± 10.1	4.8^a^ ± 6.8	5.1^b^ ± 7.2	5.7^c^ ± 8.2	<.001
CNR	15.0^a^ ± 5.4	16.1^b^ ± 5.7	14.7^a^ ± 6.2	15.8^b^ ± 6.6	17.7^c^ ± 7.5	<.001

Data are presented as mean ± standard deviation. The same superscript refers to statistically insignificance (alphabetic order refers to the order starting from the lowest mean value). AA = ascending aorta, AV-80 and AV-100 = ASIR-V with blending factors of 80% and 100%, respectively, CNR = contrast-to-noise ratio, DA = descending aorta, HU = Hounsfield units, LA = left atrium, LPA = left pulmonary artery, LV = left ventricle, PT = pulmonary trunk, RA = right atrium, RPA = right pulmonary artery, RV = right ventricle, SD = standard deviation, SNR = signal-to-noise ratio, and TF-L, TF-M, and TF-H = TrueFidelity with low, medium, and high strength levels, respectively. *P* values were calculated using repeated measures ANOVA among the 5 groups.

Comparing AV-100 and TF-H with the lowest noise and highest SNR among ASIR-V and TF reconstruction images, the image noise of TF-H was significantly lower than that of AV-100 in all chambers and vessels (*P* < .01). Regarding comparison of CNR, TF-H was also significantly better than AV-100 (*P* < .01).

### 3.2. Subjective analysis

Table 2 shows the results of the subjective analysis. In the case of quantum mottle in both readers, the TF-L score was the highest (1.1 ± 0.5) (*P* < .01). After subjectively assessing the degree of quantum mottle and blurring, TF-H scored the lowest among the image sets examined (*P* < .01). Inter-reader reliability was excellent (κ = 0.93–0.94).

**Table 2:** Subjective analysis.

Parameter	Reader 1					Reader 2					κ
	AV-80	AV-100	TF-L	TF-M	TF-H	AV-80	AV-100	TF-L	TF-M	TF-H	
Mottle	0.6 ± 0.6	0.3 ± 0.5	1.1 ± 0.5	0.6 ± 0.5	0.3 ± 0.4	0.7 ± 0.5	0.4 ± 0.5	1.1 ± 0.5	0.6 ± 0.5b	0.2 ± 0.4	0.94
Blurring	0.5 ± 0.5	1.1 ± 0.5	0.3 ± 0.5	0.2 ± 0.4	0.1 ± 0.3	0.6 ± 0.5	1.1 ± 0.4	0.3 ± 0.4	0.2 ± 0.4	0.1 ± 0.3	0.93
Overall	1.2 ± 0.8	1.5 ± 0.8	1.4 ± 0.8	0.8 ± 0.7	0.4 ± 0.6	1.2c ± 0.8	1.4 ± 0.7	1.4 ± 0.7	0.8 ± 0.7	0.4 ± 0.6	

Data are presented as mean ± standard deviation. Inter-reader reliability (κ) was evaluated for qualitative analysis between readers by using linear-weighted kappa statistics. Two radiologists independently evaluated quantum mottle and blurring by using a 4-grade system: quantum mottle (grade 0, diffuse homogeneous without mottle; grade 1, mild mottle; grade 2, moderate mottle; and grade 3, diffuse inhomogeneous with severe mottle) and blurring (grade 0, no blurring; grade 1, mild blurring; grade 2, moderate blurring; and grade 3, severe blurring). Overall image quality was defined as the sum of the quantum mottle and blurring grades. AV-80 and AV-100 = ASIR-V with blending factors of 80% and 100%, respectively. TF-L, TF-M, and TF-H = TrueFidelity with low, medium, and high strength levels, respectively.

### 3.3. Radiation dose

The average radiation dose was 0.44 ± 0.18 mSv (radiation dose range: 0.15–1.01 mSv).

## 4. Discussion

In this study, we compared pediatric cardiac CTA images reconstructed by conventional IR with 2 blending factors of 80% and 100% (AV-80 and AV-100) and DLR with 3 strength levels (TF-L, TF-M, and TF-H). According to our objective and subjective analyses, ASIR-V improved SNR and CNR when the blending factor increased. However, there was a dilemma in the quality of the image because of the disadvantage of blurring. On the contrary, when the strength of TF increases, noise, image mottle, and blurring decrease, thus improving the overall image quality.

When comparing ASIV-100 and TF-H, which have best image quality among each reconstruction method, the noise and SNR of TF-H were significantly lower and higher than those of AV-100, respectively. Regarding CNR, TF-H was significantly better than AV-100. In addition, in the subjective analysis assessing the degree of quantum mottle and blurring, TF-H scored the lowest among all the image sets examined. Consequently, TF-H is the best image set for denoising and improving image texture.

These days, cardiac CTA plays an important role in the diagnosis and treatment of CHD in children^[1,2]^ because it is necessary for the surgeon to preoperatively plan and prevent postoperative complications.^[7]^ In particular, considering that most cases of CHD are examined prenatally, cardiac CTA is evaluated immediately after birth. Furthermore, cardiac CTA is repeatedly performed during the postoperative period. Therefore, reducing radiation exposure in pediatric patients with CHD under 1 year of age is challenging.

Pediatric cardiac CT was developed to maintain image quality while reducing radiation exposure. First, to reduce the radiation dose itself, ECG-gated CT^[28]^; partly given the higher radiation exposure at specific portion of cardiac cycle, and automatic tube current/voltage selection; automatic selection of kVp and mA settings that give images of specified CNR, so it can reduce unnecessary radiation exposure.^[7]^ The use of various image reconstruction algorithms can improve the image quality as much as possible.^[29]^ The images reconstructed by the most widely used IR algorithm show good qualities and low radiation exposure in various studies.^[11,12]^ However, these images show an unfamiliar visual appearance to radiologists,^[13–15]^ thus adversely affecting image readings.

To compensate for these shortcomings, various vendors have developed some deep learning-based image reconstruction techniques that introduces a deep convolutional neural network, including TF. In a phantom study, DLR reduced noise and improved spatial resolution and detectability without a perceived alteration of the texture, which is commonly reported with IR.^[22]^ According to many studies, image noise, overall image quality, SNR, and CNR improve with DLR compared with conventional IR in most anatomic sections,^[17,18,21,24]^ including cardiac and coronary CTA.^[20,30]^

In IR, the human engineer or scientist should optimize the imaging parameters, So IR process limits the number of parameters to be manually optimized. In contrast, DLR can handle complex models and a number of parameters far beyond the abilities of human, so could create the better images by its training process using the high quality FBP images data set taken with high dose radiation.^[16]^

When DLR was applied to the pediatric population by using a relatively lower kVp, it improved image quality and dose reduction without sacrificing noise texture and spatial resolution.^[17]^ In addition to image quality, DLR shows better object-detection accuracy and radiologist confidence.^[19]^

In our study, an increase in noise is inevitable because cardiac CTA was performed using a very low tube voltage of 70 kVp for pediatric patients under 1 year of age. Therefore, our aim is to determine which reconstruction method will maintain image attenuation or image texture while reducing noise.

In summary, according to our study with DLR reduces increases SNR while reducing noise at a low kVp compared to ASIV, and uncomfortable image texts (plastic like, paint-brush, blotchy, oroversmooth) resulting from the image reconstructed result are the most unreacted currently. Our study has several limitations. First, this was a retrospective single-center study. Second, this study only performed a comparative evaluation of image quality; therefore, actual clinical accuracy could not be explained. Further research is needed to provide evidence of diagnostic and therapeutic benefits for clinicians. Third, AV and TF are from vendor-specific IR (GE); therefore, our results are not generally applicable to different generations of IR from different vendors. Lastly, because our study was conducted on children under the age of 1 year old, all patients was sedated to achieve optimal imaging with minimizing motion artifacts

## 5. Conclusion

Image noise, SNR, CNR, and image texture confirmed that TF-H was a better image set than AV-100 in the pediatric cardiac CTA images of patients with CHD.

This study was supported by a grant from Taejoon Pharmaceutical Co., Ltd.

This study was also supported by a 2021 research grant from Pusan National University Yangsan Hospital.

## Author contributions

**Writing – original draft:** Kun Hee Kim, Ki Seok Choo, Kyoung Jin Nam, Kyeyoung Lee, Jae-Yeon Hwang, ChanKue Park, Woo Jung Yang.
